# Supplementary material for: P2Y12 Inhibitor Monotherapy versus Conventional Dual Antiplatelet Therapy in Patients with Acute Coronary Syndrome after Percutaneous Coronary Intervention: A Meta-Analysis
Source: Pharmaceuticals (Basel). 2023 Feb 3;16(2):232. doi: 10.3390/ph16020232 (PMC9958698; doi:10.3390/ph16020232)
Supplement: Supplementary file 1 [file pharmaceuticals-16-00232-s001.zip › pharmaceuticals-2081964-supplementary.pdf]

Supplemental Table 1. EMBASE search strategy.

- The Embase database: Contains biomedical literature from 1974 to present.
- The MEDLINE & PubMed database: Covers journals from 1966 to present.
- Embase Classic: The Embase back file covering almost 2 million biomedical and pharmacological citations drawn from over 3,000 international titles from between 1947 and 1973

Supplemental Table 2. PubMed search strategy.

Search	Query	Results
#1	'percutaneous coronary intervention':ab,ti OR 'percutaneous coronary revascularization':ab,ti OR pci:ab,ti OR (('percutaneous coronary' NEAR/3 intervention):ab,ti) OR (('percutaneous coronary' NEAR/3 revascularization):ab,ti) OR 'drug eluting stents':ab,ti OR 'drug-eluting stents':ab,ti OR des:ab,ti	153,917
#2	'coronary artery disease':ab,ti OR 'myocardial infarc*':ab,ti OR 'acute coronary syndrome':ab,ti OR acs:ab,ti OR cad:ab,ti OR 'cardiovascular event':ab,ti	511,883
#3	#1 AND #2	62,353
#4	'dual antiplatelet therapy':ab,ti OR 'dual anti-platelet therapies':ab,ti OR 'dapt'/exp OR dapt	13,138
#5	'monotherapy with p2y12 inhibitor':ab,ti OR 'p2y12 inhibitor monotherapy':ab,ti OR 'shot term p2y12 inhibitor':ab,ti OR 'shot-term p2y12 inhibitor':ab,ti OR clopidogrel:ab,ti OR prasugrel:ab,ti OR ticagrelor:ab,ti OR 'clopidogrel monotherapy':ab,ti OR 'ticagrelor monotherapy':ab,ti	29,152
#6	#4 AND #5	4,983
#7	#3 AND #6	1,809
#8	'clinical trial'/de OR 'randomized controlled trial'/de OR 'randomization'/de OR 'single blind procedure'/de OR 'double blind procedure'/de OR 'crossover procedure'/de OR ('randomized controlled' NEXT/1 trial*) OR rct OR 'randomly allocated' OR 'allocated randomly' OR 'random allocation' OR (allocated NEAR/2 random) OR (single NEXT/1 blind*) OR (double NEXT/1 blind*) OR ((treble OR triple) NEAR/1 blind*)	1,922,855
#9	'meta analysis':ab,ti OR 'systematic review':ab,ti OR sr:ab,ti	487,729
#10	'net work':ab,ti OR network:ab,ti	575,436
#11	#9 OR #10	1,046,092
#12	#7 AND #8 NOT #11	462

#	Searches	Results
**#1**	"percutaneous coronary intervention"[Mesh] OR "coronary intervention*, percutaneous":[tiab] OR "intervention*, percutane-ous coronary":[tiab] OR "pci":[tiab] OR "percutaneous coronary revascularization":[tiab] OR "coronary revascularization*, percu-taneous":[tiab] OR "percutaneous coronary revascularizations":[tiab] OR "revascularization*, percutaneous coronary":[tiab] OR "drug eluting stent*":[tiab] OR "des":[tiab]	**100,646**
**#2**	"Dual Anti-Platelet Therapy"[Mesh] OR "dual antiplatelet therapy":[tiab] OR "dapt":[tiab] OR "aspirin":[tiab] OR "clopidogrel":[tiab] OR "prasugrel":[tiab] OR "ticagrelor":[tiab] OR "p2y12 inhibitor":[tiab] OR "anti-platelet therapies, dual":[tiab] OR "anti-platelet therapy, dual":[tiab] OR "dual anti-platelet therapy":[tiab] OR "dual anti-platelet therapies":[tiab]	**66,072**
**#3**	"Randomized Controlled Trial"[Publication Type] OR "controlled clinical trial":[tiab] OR "clinical trials, randomized":[tiab] OR "trials, randomized clinical":[tiab] OR "randomized clinical studies":[tiab]	**581,413**
**#4**	"Systematic Review"[Publication Type] OR "Meta-Analysis" [Publication Type] OR "Review"[Publication Type] OR "meta-analysis":[ti] OR "systematic review":[ti] OR "literature review" :[ti] OR "expert consensus":[ti] OR "case report":[ti]	**3,448,440**
**#5**	**#1 AND #2 AND #3 NOT #4**	**1,157**

Supplemental Table 3. Cochrane Library search strategy.

#	Searches	Results
**#1**	MeSH descriptor percutaneous coronary intervention explode all trees OR (coronary intervention*, percutaneous):ti,ab,kw OR (intervention*, percutaneous coronary):ti,ab,kw OR (pci):ti,ab,kw OR (percutaneous coronary revascularization*): ti,ab,kw OR (coronary revascularization*, percutaneous):ti, ab,kw OR (revascularization*, percutaneous coronary):ti,ab, kw OR (drug eluting stent*):ti,ab,kw OR (drug-eluting stent): ti,ab,kw OR (des):ti,ab,kw	**16,722**
#2	(dual antiplatelet therapy):ti,ab,kw OR (dapt):ti,ab,kw OR (aspirin):ti,ab,kw OR (clopidogrel):ti,ab,kw OR (prasugrel):ti, ab,kw OR (ticagrelor):ti,ab,kw OR (p2y12 inhibitor):ti,ab,kw OR (anti-platelet therapies, dual):ti,ab,kw OR (anti-platelet therapy, dual):ti,ab,kw OR (dual anti-platelet therapy):ti,ab, kw OR (dual anti-platelet therapies):ti,ab,kw	**19,281**
**#3**	'MeSH descriptor randomized controlled trial explode all trees OR (controlled clinical trial):ti,ab OR (clinical trials, randomized):ti,ab OR (trials, randomized clinical):ti,ab OR (randomized clinical studies):ti,ab	**224,589**
**#4**	**#1 AND #2 AND #3**	**670**

	Random sequence generation (selection bias)	Allocation concealment (selection bias)	Blinding of participants and personnel (performance bias)	Blinding of outcome assessment (detection bias)	Incomplete outcome data (attrition bias)	Selective reporting (reporting bias)	Other bias
GLOBAL LEADER ACS	+	+	−	+	+	+	?
SMART CHOICE	+	+	−	+	+	+	+
STOPDAPT2-ACS	+	+	−	+	+	+	+
TICO	+	+	−	+	+	+	+
TWILIGHT-ACS	+	+	+	+	+	+	+

Supplemental Table 4. Bias risk assessment of the studies.

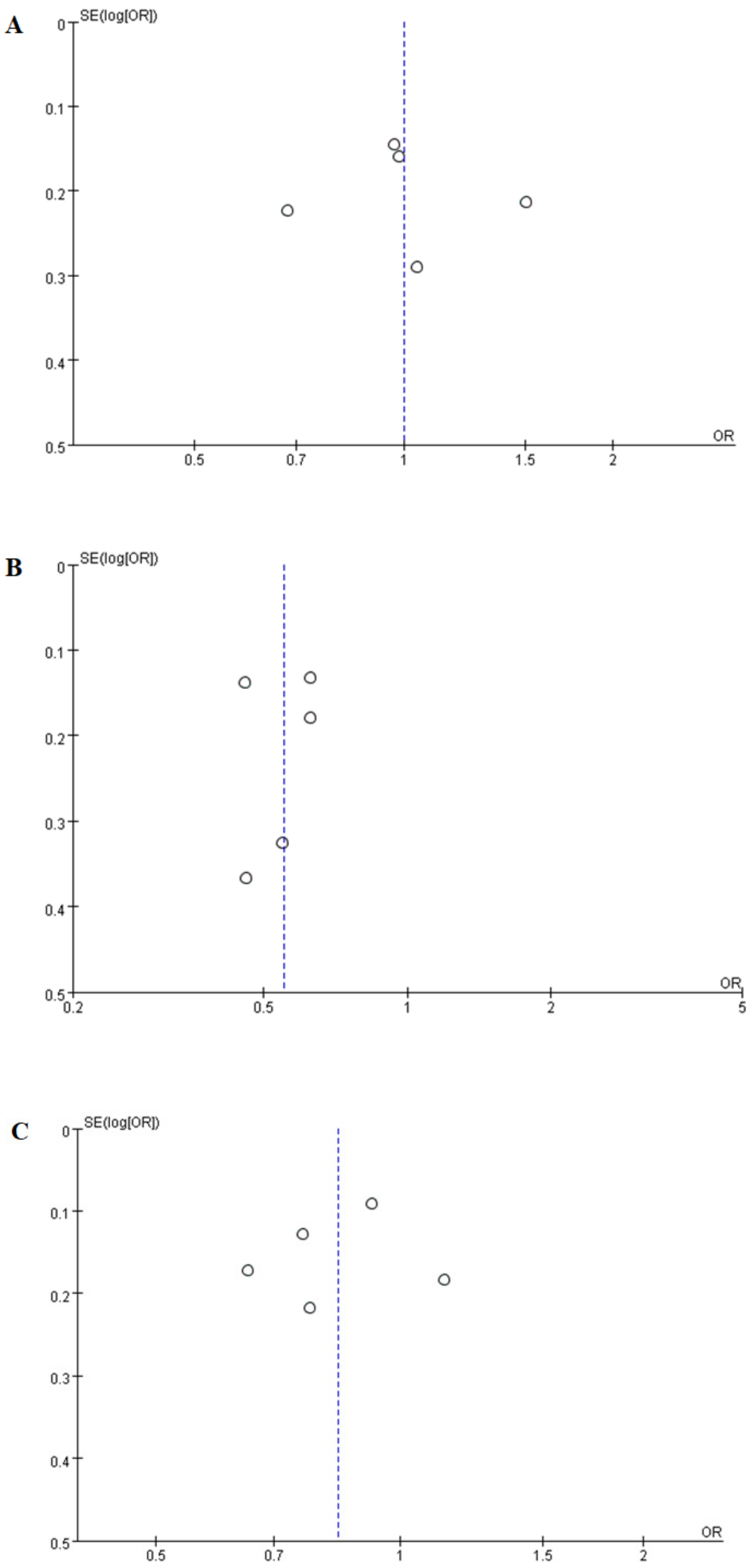

Supplemental Figure S1. The funnel plots of each outcome. (A) MACCE, (B) major or minor bleeding, (C) NACE.
